# Electromyographically controlled prosthetic wrist improves dexterity and reduces compensatory movements without added cognitive load

**DOI:** 10.1038/s41598-024-73855-1

**Published:** 2024-10-06

**Authors:** Connor D. Olsen, Nathaniel R. Olsen, Eric S. Stone, Troy N. Tully, Michael D. Paskett, Masaru Teramoto, Gregory A. Clark, Jacob A. George

**Affiliations:** 1 Department of Electrical Engineering, University of Utah, Salt Lake City, USA; 2 Department of Mechanical Engineering, University of Utah, Salt Lake City, USA; 3 Department of Biomedical Engineering, University of Utah, Salt Lake City, USA; 4 Department of Physical Medicine and Rehabilitation, University of Utah, Salt Lake City, USA

**Keywords:** Prosthesis, Wrist, Compensation, Cognitive load, Biomedical engineering, Motor control

## Abstract

Wrist function is a top priority for transradial amputees. However, the combined functional, biomechanical, and cognitive impact of using a powered prosthetic wrist is unclear. Here, we quantify task performance, compensatory movements, and cognitive load while three transradial amputees performed a modified Clothespin Relocation Task using two myoelectric prostheses with and without the wrists. The two myoelectric prostheses include a commercial prosthesis with a built-in powered wrist, and a newly developed inexpensive prosthetic wrist for research purposes, called the “Utah wrist”, that can be adapted to work with various sockets and prostheses. For these three participants, task failure rate decreased significantly from 66% ± 12% without the wrist to 39% ± 9% with the Utah wrist. Compensatory forward leaning movements also decreased significantly, from 24.2° ± 2.5 without the wrist to 12.6° ± 1.0 with the Utah wrist, and from 23.6° ± 7.6 to 15.3° ± 7.2 with the commercial prosthesis with an integrated wrist. Compensatory leftward bending movements also significantly decreased, from 20.8° ± 8.6 to 12.3° ± 5.3, for the commercial with an integrated wrist. Importantly, simultaneous myoelectric control of either prosthetic wrist had no significant impact on cognitive load, as assessed by the NASA Task Load Index survey and a secondary detection response task. This work suggests that functional prosthetic wrists can improve dexterity and reduce compensation without significantly increasing cognitive effort. These results, and the introduction of a new inexpensive prosthetic wrist for research purposes, can aid future research and development and guide the prescription of upper-limb prostheses.

**Supplementary Information:**

The online version contains supplementary material available at 10.1038/s41598-024-73855-1.

## Introduction

Transradial amputees have expressed a strong desire for powered wrist prostheses. Indeed, the top five priorities for transradial amputees, in order of importance, were reported as: wrist rotation, simultaneous movements of multiple degrees of freedom, wrist deviation, wrist flexion/extension, and reduced cognitive demand. Other priorities included reduced weight, improved durability, and increased strength^1,2^. Without a wrist, amputees are forced to compensate with unnatural movements to complete routine activities of daily living (ADLs)^3,4^. The continual use of these motions causes damage to the musculoskeletal system over time^5,6^.

Despite end-user desire for functional wrist movements, only a few prostheses incorporate a powered wrist module, and those that do or either financially unobtainable or unavailable altogether^7,8^. Many prostheses can be paired with a powered wrist, but commercially available options are few and are often limited to a single active degree of freedom (DOF)^9–11^. Passive wrists often have additional DOFs, but these are unnatural to use, as they require manual adjustment with the contralateral hand, and manual adjustments are particularly inefficient for unilateral tasks. Some commercially available hands come with their own powered wrist, but these are often expensive, heavy, or not widely available.

In response to this, there have been several publications showcasing new 3D-printed wrists^9,12–16^. 3D-printed wrists have been designed to enable simultaneous active control of multiple DOFs but have often stopped short of validation with amputee end-users^12–14^ or are limited to only a case study with a single participant^9,16^. Importantly, many of these 3D-printed wrists are motivated by the desire to reduce compensatory movements^9,12^, but they have yet to quantify the impact of using an active wrist on compensatory movements.

Building on these prior works, here we describe the development of a powered, 3D-printed, inexpensive, and adaptable prosthetic wrist for research purposes, referred to herein as the “Utah wrist.” The Utah wrist has a unique modular design which allows it to easily attach to different sockets and terminal devices, making it a practical and cost-effective tool to aid future prosthetic wrist research. Importantly, we validate the function of the Utah wrist with three transradial amputees and show comparable performance of the Utah wrist to that of a high-end commercially available wrist in a research setting.

More importantly, we introduce several new metrics for validating use of a prosthetic wrist. First, we show that use of a powered prosthetic wrist reduces compensatory leftward bending and forward leaning motions using wearable inertial measurement units (IMUs). Second, we show that adding simultaneous myoelectric control of the wrist does not require more cognitive demand using a survey of subjective workload and a novel secondary detection response task of cognitive load (DRT). Together, these findings and the development and validation of a new prosthetic wrist constitute an important step toward addressing amputees’ self-reported needs and reducing compensatory movements that would otherwise cause musculoskeletal damage.

## Methods

### Wrist design

**Fig. 1:**
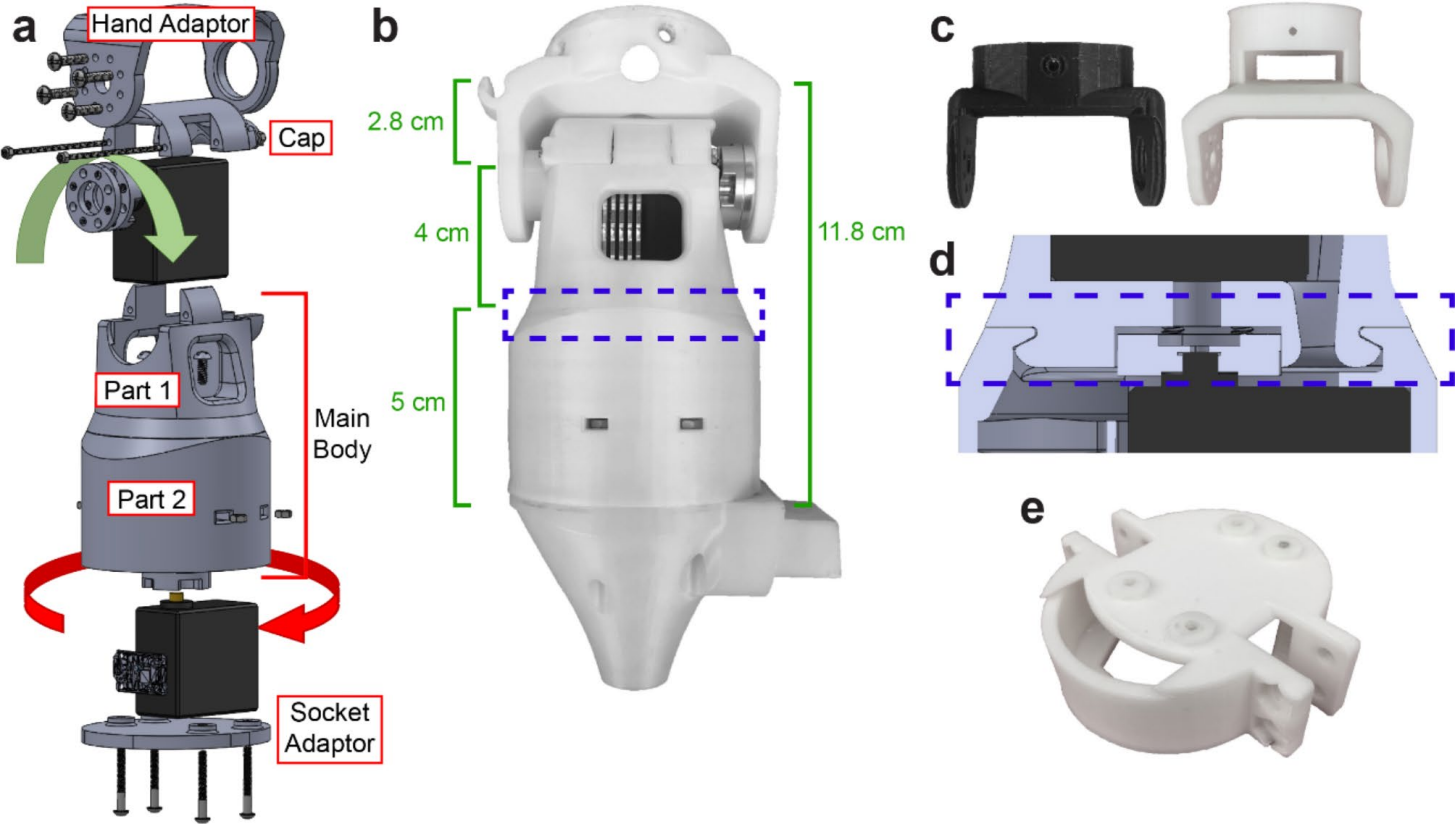
Design of the Utah wrist. **a** Exploded view of the Utah wrist. The red and green arrows correspond to the motors associated with pronation/supination and flexion/extension (or ulnar/radial deviation), respectively. **b** Photo and dimensions of the assembled wrist with the attachments to connect to a bypass socket. **c** The wrist can adapt to various terminal devices by printing a new interface part such as the two shown here. **d** Expanded view of the rotary joint mechanism, as highlighted in part **b**. **e** The wrist can connect to various sockets by printing a new interface part, such as the one shown here.

The Utah wrist was designed with two degrees of freedom (DOFs) to provide pronation/supination and flexion/extension (Fig. 1). The second DOF can also be used for deviation depending on how the prosthesis is mounted to the wrist. The body of the wrist consists of an interlocking rotation mechanism and cap. The interlocking rotation mechanism was designed to be 3D-printed with dissolvable support material, polyvinyl alcohol (PVA), between the interlocking parts (Fig. 1a). The two subparts are printed together as a single piece and can freely rotate once the inner support material is dissolved (Fig. 1b and d) This design and assembly process strengthens the wrist, resulting in a more robust device that can support the weight of a terminal device. Each of these subparts houses a servo motor, and the proximal subpart also houses an Adafruit Trinket M0 microcontroller (Adafruit Industries, New York, NY). The two high-power hobby servo motors (Hitec D980TW, Hitec RCD USA, Poway, CA) were chosen to mimic the strength of a human wrist and a 7.5-V, 20-A power supply (967-CUS200LD7R5, TDK-Lambda Americas Inc., National City, CA) was used to provide 4.3 N-m of torque. An Actobotics 525,130 servo hub horn (RobotZone, Winfield, KS) was mounted to the proximal motor, then fixed to the distal part via four screws. The distal servo motor was responsible for the additional degree of freedom (flexion/extension or radial/ulnar deviation) and had a servo hub horn mounted to it. The servo hub horn is fixed to the adaptable hand attachment portion of the wrist using four screws. Actobotics 545,372 servo hub spacers (RobotZone, Winfield, KS) provided proper spacing between the servo horn and the adaptable hand attachment portion of the wrist.

The average length of the radius is 24.6 cm (SD = 1.25) for males and 22.0 cm (SD = 1.03) for females^17^. The Utah wrist measures 11.8 cm (Table 1), making it less than half the average length of a male forearm and only 0.8 cm longer than half the average length of a female forearm. Minimizing the length of the Utah wrist was a top priority as residual limb lengths can vary. Minimizing the length also constrained the design to two motors and thus only a 2-DOF wrist. Pronation/supination was included in the design because it is the most frequently implemented DOF^18^. The Utah wrist was designed to operate in series with either flexion/extension or radial/ulnar deviation: either can be selected by mounting the wrist in its standard configuration or at a 90º offset to the hand. This configuration is changed by physically removing and remounting the hand to the wrist. For our validation study, we chose to use the flexion/extension configuration to match the capability of the high-end commercial wrist we assessed in parallel. Additional details on the commercial wrist are provided in the “Functional assessment” section.

The Utah wrist was built to accommodate different prostheses and sockets using modular attachments. CAD design and 3D printing allow for rapid adaptation to the distal and proximal ends of the device (Fig. 1e) and to various prosthetic hands (Fig. 1c). The distal hand adaptor was designed with a flat surface to easily print modified attachments to attach new prostheses to the Utah wrist. Likewise, the proximal end was designed to be easily modified to fit various sockets. The Utah wrist was 3D printed using polylactic acid (PLA) to minimize the weight and cost of materials. See Table 1 for the full design specifications.

**Table 1:** The wrist specifications fall within the established design criteria.

Wrist specifications	
Degrees of freedom	Pronation/supination in series with flexion/extension or radial/ulnar deviation
Length	11.8 cm
Weight	360 g
Range of motion	Pronation/supination – 180 Degrees Radial/ulnar deviation or flexion/extension – Up to 175 Degrees
Torque	4.3 N*m
Cost	< $600

### Human subjects

We recruited three male transradial amputees between the ages of 45 and 60. Residual limb lengths were between 7.6 and 17.8 cm. Due to only left-hand prostheses availability, only left transradial amputees were recruited. All participants had prior myoelectric control experience, though only one used a myoelectric device for daily use. The two others preferred a cosmetic hand and body-powered hook respectively. Individual participant demographics have been included in Table 2. All participants gave written informed consent before taking part in experiments. All methods were conducted in accordance with the University of Utah Institutional Review Board (IRB 00098851), the Department of Navy Human Research Protection Program, and the Declaration of Helsinki. Written consent was obtained to publish photos.

**Table 2:** Participant demographics.

Participant	Sex	Age	Preferred Prosthesis	Hand Dominance	Years Since Amputation
1	M	60	Cosmetic Hand	Left	20
2	M	44	Body Powered Hook	Right	12
3	M	55	Taska Hand	N/A	N/A

### Functional assessment

**Fig. 2:**
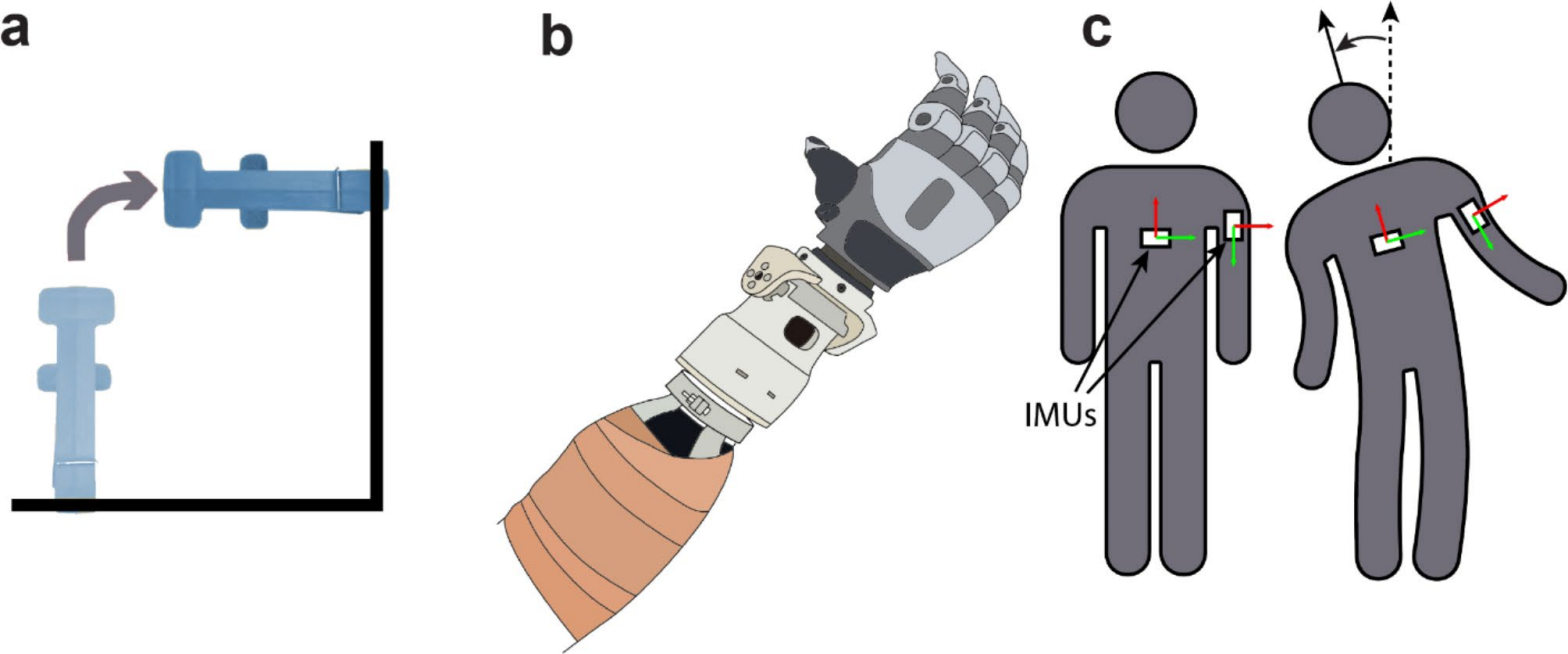
Methods **a** The amputee participants were instructed to pick up a clothespin and move it from a horizontal beginning position to a vertical end position. **b** The Utah wrist was attached to the amputee participants using a multi-user functional check socket^20^. **c** IMUs were attached to the amputee participant’s chest and bicep to measure the compensatory motions when attempting to complete the task. The white blocks represent the placement of the IMUs and their change in orientation while the participant performed the task. See Supplementary Figure S1 for a photograph of one participant completing the task.

The clothespin relocation task (CRT) is a commonly used upper-limb dexterity assessment that involves moving a clothespin from a horizontal bar to a vertical bar. The clothespin is placed 20 cm down the length of the horizontal bar and 20 cm up the vertical bar (Fig. 2a & Supp. Fig. S1). Participants completed a modified version of the clothespin relocation task found in^19^ where the number of successful completions is recorded as the performance metric. Participants were instructed to move as many clothespins as possible in a 30-second window. The total number of attempts and the percentage of successful attempts were recorded. An attempt was recorded as unsuccessful if the participant removed the clothespin from its initial position but dropped it before placing it on the vertical bar. The CRT was completed by the three transradial amputee participants with two prosthesis configurations. The first configuration, herein referred to as the research configuration (Supp. Fig. S2), involved the new Utah wrist affixed to a commercial prosthetic hand (TASKA Prosthetics, Christchurch, New Zealand). The research configuration had a total weight of 1100 g, and was ~ 36.9 cm from socket to fingertip. The second configuration, herein referred to as the commercial configuration, involved a commercial prosthetic arm with a built-in powered wrist (LUKE Arm, Mobius Bionics, Manchester, New Hampshire, USA). The commercial configuration had a total weight of 1500 g and was ~ 31.8 cm from socket to fingertip. The weights provided for the prosthesis configurations does not include the battery, as both configurations were powered using an external battery that was not affixed to the arm. The commercial configuration (LUKE Arm) uses a battery pack attached to the hip; similarly, our research configuration (Utah Wrist + TASKA Hand) was powered using an external battery. Participants were given two blocks of 30 s to complete the CRT under four conditions: (1) commercial configuration with wrist disabled (C-W), (2) commercial configuration with wrist enabled (C+W), (3) research configuration with Utah Wrist disabled (R-W), and (4) research configuration with Utah Wrist enabled (R+W). The participants donned both prostheses using a multi-user functional check socket^20^, which is shown with the research configuration in Fig. 2b. Data were collected under a pseudo-randomized counter-balanced cross-over design to minimize order effects such as learning and fatigue. The failure rate was defined as the total number of unsuccessful transfers out of the total number of attempts within the 30-second time period.

### Myoelectric control

Most commercial prostheses provide only a single DOF that is controlled using conventional dual-site control^21^. Adding a wrist, as done in this study, requires an individual to control three DOFs (i.e., wrist rotation, wrist flexion/extension, and hand open/close) instead of just one. Traditionally, commercial myoelectric prostheses with multiple DOFs are controlled using pattern recognition^22^. However, prior work has shown that simultaneous myoelectric control of these three degrees of freedom outperforms sequential pattern recognition control for tasks requiring multiple degrees of freedom^23^. Since the CRT requires both hand and wrist movement, we provided the participants with simultaneous control over the hand and wrist DOFs using high-density EMG^24^ and simultaneous regression^25^, which has been shown to computationally efficient^26^ and highly effective for activities of daily living^27^. Details regarding this control strategy are described in the following sections.

### Signal acquisition

Surface Electromyographic (sEMG) recordings were sampled at 1 kHz using the Grapevine Neural Interface Processor (Ripple Neuro LLC, Salt Lake City, UT, USA). Continuous EMG signals (32 channels) were band-pass filtered with cutoff frequencies of 15 Hz (sixth-order high-pass Butterworth filter) and 375 Hz (second-order low-pass Butterworth filter). Notch filters were also applied at 60, 120, and 180 Hz. Differential EMG signals were calculated for all possible pairs of channels, resulting in 496 (32 choose 2) differential pair recordings. The mean absolute value (MAV) was then calculated for the 32 single-ended recordings and the 496 differential recordings at 30 Hz. The MAV was smoothed using an overlapping 300-ms window. The resulting EMG feature set consisted of the 300-ms smoothed MAV on 528 channels (32 single-ended channels and 496 differential pairs), calculated at 30 Hz, as shown previously in^25^.

### Prosthetic control training

EMG was recorded while amputees mimicked pre-programmed movements of the prosthetic hand and wrist. The pre-programmed movements consisted of four repetitions of the following motions: opening and closing a pinch grip, pronating and supinating the wrist, and flexing and extending the wrist. The movements also consisted of two repetitions of each possible paired sequential combination of hand and wrist motion (e.g., pinch followed by pronation, pronation followed by pinch, pinch followed by supination, supination followed by pinch)^27^.

### Machine-learning algorithm

The acquired sEMG data were used to train a modified Kalman filter (mKF)^25^ which provided the participants continuous, proportional control of the three trained DOFs: pinching, pronating/supinating the wrist, and flexing/extending the wrist. The mKF provides an efficient recursive algorithm to optimally estimate the position of the bionic hand when the likelihood model (i.e., the probability of EMG activity given the current kinematic position) and prior models (i.e., the state model of how kinematics change over time) are linear and Gaussian^28^. The inclusion of prior information about the system state enables an efficient recursive formulation of the machine-learning algorithm and effectively smooths noisy estimates in a mathematically principled way^29^. The same mKF was used for all four experimental conditions. For conditions without a wrist (i.e., C-W and R-W), the two DOFs associated with the wrist were locked in software, thereby ensuring the participants’ control remained the same across all conditions.

### Body compensation

Joint angles were measured using three Shimmer3 Inertial Measurement Units (IMUs) (Shimmer Sensing, Dublin, Ireland) attached to the participant’s chest and bicep (Fig. 2c). A third IMU was placed on the table in a fixed orientation to provide a reference. The IMUs measured acceleration, rate gyration, and magnetic heading at 64 Hz, which were then used to calculate quaternions and generate rotation matrices for forward lean and leftward bend at the hip. Compensation was measured as the maximum of the absolute value of the angle deviation during an attempted movement. Maximum angle deviation for leftward bend at the hip has been shown to increase for amputees compared to non-amputee controls^3^.

### Cognitive load

While completing the CRT, the participants simultaneously completed a detection-response task (DRT) to measure their cognitive load^30^. The DRT was devised as a method for measuring the attentional effects of cognitive load in a driving environment^30–33^ and has recently been shown to measure cognitive load while operating a prosthesis^34–36^. The DRT requires the participant to push a button in response to a vibrating stimulus on their collarbone. The vibration stimulations were delivered randomly every two to four seconds, resulting in five to eight stimulations during each 30-second window. Both the response miss rate (i.e., how often they failed to respond to the vibratory stimuli) and response time (i.e., how long it takes to press the button after vibratory stimuli) were used as measures of cognitive load. Responses over 2.5 s were considered misses and excluded from further analyses regarding the response time, according to the ISO standard^32^. Response rates are bound by two extremes: (1) a 0% miss rate in which there is enough available cognition devoted to the secondary task to achieve perfect performance, and (2) a 100% miss rate in which there is no cognition available for the secondary task. After each wrist configuration, participants also completed the NASA Task Load Index (TLX), a self-assessment of subjective workload scored between zero (easy) and 100 (difficult). The minimum change for the NASA TLX score to be attributed to true change (beyond measurement error) has been reported as 15 points^37^.

### Data analysis

Participants were given two blocks of 30 s to complete the CRT as many times as possible for a given condition. Thus, the number of attempted clothespin transfers varied per condition and participant. A total of seven outcome variables were analyzed: (1) Success Rate (dichotomous variable reported as proportion), (2) Leftward Bend (continuous variable), (3) Forward Lean (continuous variable), (4) Attempted Movements (continuous variable), (5) Subjective Workload (continuous variable), (6) Response Time (continuous variable), and (7) Response Accuracy: Accuracy (dichotomous variable reported as proportion). There were two sets of conditions for comparisons (= independent variables): (1) C+W vs. C-W and (2) R+W vs. R-W. First, unadjusted (raw) means and standard deviations (SD) were calculated for these variables. Then, an appropriate, multivariate statistical model was fit to the data on each outcome variable. Specifically, a generalized linear model (GLM) with binomial distribution and log link^38,39^ was used to examine Success Rate and Response Accuracy: Accuracy. Leftward Bend, Forward Lean, and Response Time were analyzed using a linear model with the robust or sandwich estimator of variance to account for the non-normal distribution of residuals^40,41^. Further, a permutation test of mean differences, computed from the coefficient of a linear regression model, with 10,000 Monte Carlo simulations^42^, was used for Attempted Movements and Subjective Workload to make robust inferences from the smaller number of observations associated with these two variables. For all the statistical models above, participant and cycle (applicable for some outcome variables), along with condition (C+W vs. C-W or R+W vs. R-W), were included as covariates to account for correlated observations derived from repeated measures of the data. An adjusted (predicted) mean and its 95% confidence interval (CI) was calculated after building each model. All the analyses were performed using Stata/MP 18.0 (StataCorp LLC, College Station, TX), with an α level of 0.05 for statistical significance.

## Results

### Amputees required smaller compensatory movements with use of a prosthetic wrist

**Fig. 3:**
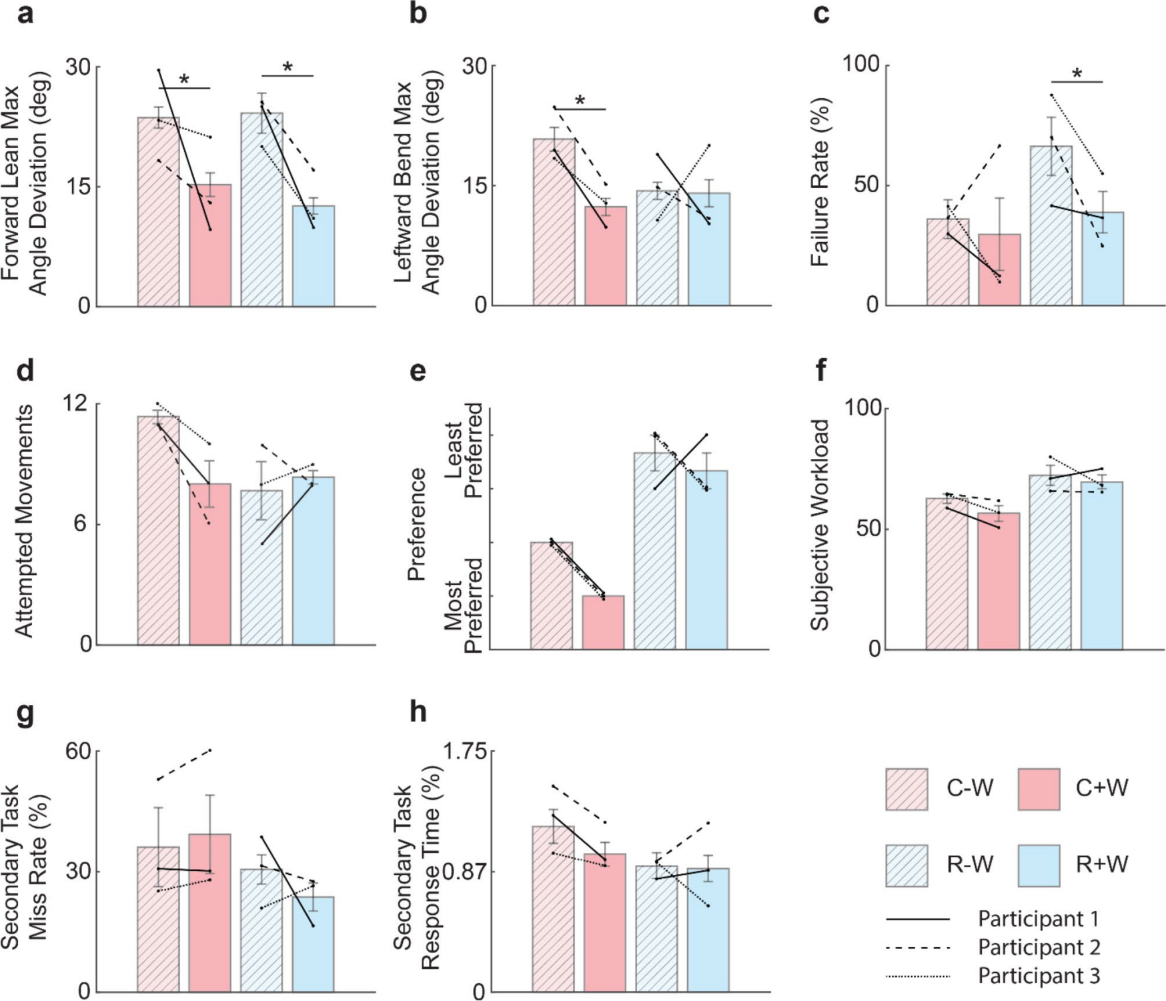
Experiment results. **a** Compensatory movement for forward lean was significantly reduced with the wrist compared to without the wrist. **b** Compensatory movement for leftward bend at the hip (i.e., the maximum angle deviation) was significantly reduced with the wrist compared to without the wrist while using the commercial prosthesis configuration only. **c** Failure rate for the clothespin relocation task (CRT) using the research prosthesis configuration decreased when the task was performed with the Utah wrist. **d** Average number of attempted movements under each condition. **e** Participants preferred to use the prostheses with the wrist enabled compared to without the wrist enabled. Note that the error bars are not present in the commercial condition, because every participant ranked the commercial condition the same. No variance between participant response is seen for the C+W and C-W condition. **f** No significant differences were seen in the subjective workload with the wrist vs. without. **g** No significant differences were seen in the DRT miss rate with the wrist vs. without. **h** No significant differences were seen in the DRT response time with the wrist vs. without. Data show mean ± standard error. Bars show aggregate data across all participants and all sessions, and lines show individual participant performance averaged across their own sessions. * *p* < 0.05. Generalized linear model with binomial distribution and log link used for Failure Rate and Secondary Task Response Accuracy. Linear model with robust or sandwich estimator of variance used for Leftward Bend, Forward Lean and Secondary Task Response Time. Permutation test of mean differences with 10,000 Monte Carlo simulations used for Subjective Workload. (*N* = 3).

Moving the clothespin from the horizontal bar to the vertical bar without the wrist required the participant to compensate by leaning backward and bending leftward at the hip. When the wrist was enabled, participants adopted a different control strategy and naturally used the wrist to perform the task with movements more akin to an intact hand. Collectively, the three participants showed a significant difference in the maximum joint angle when leaning forward, whether using the commercial prosthesis configuration or the research prosthesis configuration (Fig. 3a & Supp. Figure 3). With the commercial configuration, the maximum forward lean angle significantly decreased from 23.6° (SD = 7.6) with the wrist disabled to 15.3° (SD = 7.2) with the wrist enabled (*p* < 0.001, linear model with robust or sandwich estimator of variance). Under the research configuration, the maximum forward lean angle significantly decreased from 24.2° (SD = 12.1) without the Utah wrist to 12.6° (SD = 5.1) with the Utah wrist (*p* < 0.001, linear model with robust or sandwich estimator of variance). Adding a wrist reduced compensatory forward leaning by 35% in the commercial configuration and 48% in the research configuration.

With the commercial configuration, the maximum leftward trunk bend angle significantly decreased from 20.8° (SD = 8.6) with the wrist disabled to 12.3° (SD = 5.3) with the wrist enabled (*p* < 0.001, linear model with robust or sandwich estimator of variance; Fig. 3b). With the research configuration, the maximum hip joint angle was 14.3° (SD = 5.1) without the Utah wrist and 14.1° (SD = 8.5) with the Utah wrist (*p* = 0.88, unpaired t-test). Adding a functional wrist decreased compensatory leftward bending by 41% for the commercial configuration, while the research configuration remained unchanged.

### Task performance improved with use of wrist

In addition to promoting more natural movements, the use of a functional wrist also significantly improved performance on the CRT (Fig. 3c). However, this was significant only for the research prosthesis configuration. For the commercial configuration, the failure rate (i.e., the percentage of dropped clothespins) was 35% (SD = 0.082) with the wrist disabled and 25% (SD = 0.088) with the wrist enabled (*p* = 0.196, generalized linear model with binomial distribution and log link). For the research configuration, the failure rate decreased from 70% (SD = 0.096) without the Utah wrist to 40% (SD = 0.098) with the Utah wrist (*p* < 0.05, generalized linear model with binomial distribution and log link). In other words, performance improved by 40% for the research configuration when using the Utah wrist. Across all participants, the number of attempted movements for each condition was 34 (C-W), 24 (C+W), 23 (R-W) and 25 (R+W) (Fig. 3d).

### Most participants preferred using a prosthetic wrist

Each participant ranked the four prosthesis configurations (C-W, C+W, R-W, R+W) by preference. In general, prosthesis configurations with the wrist were preferred over their counterpart without the wrist. All three participants preferred the C+W compared to the C-W. Two of three participants preferred the R+W compared to the R-W (Fig. 3e). Overall, the commercial configuration was preferred over the research configuration, but for both configurations, adding a wrist was generally preferred.

### Use of a functional prosthetic wrist did not significantly increase cognitive load

Adding two additional controllable degrees of freedom with the wrist did not significantly increase cognitive load. When using the commercial configuration, the reported subjective workload across participants showed a decreasing, but non-significant, trend from 62.7 (SD = 3.3) with the wrist disabled to 56.6 (SD = 5.6) with the wrist enabled (*p* = 0.453, permutation test of mean differences with 10,000 Monte Carlo simulations; Fig. 3f). When using the research configuration, participants reported 72.4 (SD = 7.2) without the Utah wrist and 69.6 (SD = 5.0) with the Utah wrist (*p* = 0.740, permutation test of mean differences with 10,000 Monte Carlo simulations; Fig. 3f).

Likewise, the objective workload showed no significant increase in response time to the DRT task. Using the commercial configuration, the participants’ miss rate was 44% (SD = 0.066) with the wrist disabled and 41% (SD = 0.065) with the wrist (*p* = 0.072, generalized linear model with binomial distribution and log link; Fig. 3g). Using the research configuration, the participant miss rate was 39% (SD = 0.065) without the Utah wrist to 25% (SD = 0.059) with the Utah wrist (*p* = 0.142, generalized linear model with binomial distribution and log link, Fig. 3g).

Response time to the randomly delivered vibration stimuli also showed no significant increase when a wrist was enabled. When performing the trial with the commercial configuration, response time showed a decreasing, but non-significant, trend from 1.24 s (SD = 0.696) with the wrist disabled to 1.001 s (SD = 0.520) with the wrist (*p* = 0.073, linear model with robust or sandwich estimator of variance; Fig. 3h). Under the research configuration, response time was 0.919 s (SD = 0.545) without the Utah wrist and 0.898 s (0.605) with the Utah wrist (*p* = 0.921, linear model with robust or sandwich estimator of variance; Fig. 3h).

## Discussion

The results presented here show that using a functional prosthetic wrist decreases compensatory movements and can also improve the task rate for some prostheses. In the R+W case, the failure rate dropped significantly, though there were no significant effects for the C+W case. We speculate that one cause of this was that the research condition hand was built with fingers that could passively splay outward. This would at times cause the fingers to slip laterally when grasping the clothespin. Because the commercial condition hand had rigid fingers, it likely made the CRT easier to complete overall than the research condition, resulting in non-significant results in the C+W case over the C-W case. Compensatory movement at the hip decreased significantly for both prosthesis configurations when the wrist was enabled. Participants generally preferred to have an active wrist, regardless of which prosthesis configuration they were using. Despite the added complexity of controlling a 2-DOF wrist with electromyography, subjective and objective measures of cognitive load did not significantly increase with the added functional wrist.

Several 3D-printed prostheses with at least two DOFs have been previously developed^12–14^. For example, ^15^ shows a 2-DOF wrist that is both low profile and commercially available. Wrists have also been developed that are short in length to allow for attachment to the hand and not to be overly long and cumbersome^9,15^. High-torque designs have also been introduced to complete more demanding ADLs^14,16^. The Utah wrist builds on these previous designs by incorporating each of these key features into a single unified design that can adapt to many different sockets and terminal devices. Importantly, we also go a step further to validate the functional and cognitive benefits of the Utah wrist with amputee participants.

Joint angles have been used extensively as a measurement of compensatory movements^43–45^. Similar to^43,46^, we calculated compensatory movements by affixing IMUs to the participant’s body. For the leftward bend, our results found that the maximum joint angle deviation for the no-wrist condition ranged on average between 14 and 20 degrees. These joint angles are consistent with a previous study that measured 15–25 degrees of compensatory movement in amputees performing a similar task^3^. The same study also measured 10–12 degrees of compensatory movements in healthy, non-amputee participants performing the same task^3^. In comparison, here we measured on average between 12 and 14 degrees of compensatory movements in amputee participants using a prosthetic wrist. The similarity in compensatory movements between healthy, non-amputee participants and amputee participants using a prosthetic wrist suggests that the Utah wrist can reduce compensation to that of a healthy, non-amputee level. An additional study involving prosthesis users and able-bodied participants compares the joint angles for a similar task involving pouring liquid from a carton on the contralateral side of the body^47^. In that study, both the lateral and forward trunk flexion was reduced significantly when performed by an able-bodied user, further indicating that the decrease in compensation from using the wrist allows users to move more naturally.

We observed a 41% reduction in compensatory leftward bending for the commercial configuration, but no change in compensatory leftward bending for the research configuration. We speculate two factors may be at play here. First, we note that participants 2 and 3 had minimal compensatory leftward bending to begin with using the research configuration without the wrist (R-W). This may be attributed to differences in the prosthetic hands, as the TASKA hand used in the research configuration has a resting hand position that is slightly more conducive to grasping objects at an angle (see Supp. Fig. S1 for an example). Second, participant 3 experienced an increase in compensatory leftward bending when using the research configuration with the wrist (R+W). Factors such as socket fit or residual limb length are unlikely to cause an increase in compensation with the wrist active; socket fit should impact both conditions (R+W and R-W) equally, and residual limb length would more likely impact forward leaning than leftward bending. Instead, we suspect participant 3’s increase in compensation may be due to poor grasping control. Informal observations of participant 3’s movements suggest worse grasping control, that when coupled with the ability of the TASKA hand to grasp objects at an angle, may have resulted in suboptimal positioning of the clothespins within the prosthesis. A clothespin that partially slips from the grip of the prosthesis would likely require additional corrective compensatory movements.

The clothespin relocation task is designed to assess upper-limb dexterity. One advantage of the CRT is that it requires the participant to use more than one joint to complete, which elicits compensatory movements when those degrees of freedom are unavailable^19^. Other studies have adapted the CRT to fit their own experiments, as shown in^48^. We chose to record the success and failure of all attempts during a given time window to make full use of the DRT. Due to this choice, we are unable to make direct comparisons to the CRT scores in the literature. However, our results are still consistent with prior work that also showed that CRT performance improves with added DOFs^49^. We recognize that the control algorithm used is very specific to the CRT described herein. We had three DOFs completing a 2-DOF task, and it is unclear how EMG control of these multiple DOFs would extrapolate to more complex tasks requiring a greater number of degrees of freedom. Performance of EMG control of simultaneous degrees of freedom degrades as the number of DOFs increases^26,50^. In the case that there’s a bottleneck in the number of controllable DOFs, mode switching could be utilized for effective control to provide maximum capability. The results presented here suggest that it’s more efficient to control the hand and wrist simultaneously, which is consistent with prior work^23^. Our results expand upon prior work to also show that, at least for this control algorithm, controlling a wrist does not require additional cognitive burden and reduces compensatory movements that have been shown to cause musculoskeletal problems long term^5,6^. Though the CRT primarily required the user to use supination/pronation of the wrist to complete the task, users also used a substantial amount of wrist flexion/extension to position the hand properly to grasp the clothespins (Supp. Fig. S4). In fact, the participants on average used more flexion and extension of the wrist than supination and pronation (Supp. Fig. S5). The differences in the total range of motion between the C+W and R+W conditions may be attributed to prosthesis length and fit.

Because the objective of this study was to explore how having a wrist impacts cognitive and functional performance, we selectively created a single algorithm for all three to eliminate variability in the controller between conditions. That said, we recognize that having a controller specific to a lesser number of DOFs could result in better performance for the 1-DOF condition and 2-DOF configurations. However, given that the participants were able to complete the task successfully under the 3-DOF condition, and the primary differences were in cognitive load and joint angle compensation, we don’t suspect that minor increases in control algorithm function for the subset of degrees of freedom would outweigh the benefit of not having to compensate.

NASA’s TLX survey has been used extensively to subjectively measure cognitive load when working with prostheses and EMG control^51–53^. We expanded on this idea by adding an objective measure of cognitive load via the DRT. This combination is designed to give a more holistic view of the cognitive workload experienced by the participants. Our NASA-TLX scores ranged from 60 to 76 on a hundred-point scale on the no-wrist condition. A similar study using the TASKA hand for a pinching grasp reported numbers between 60 and 80. This suggests a base level of cognitive weight for simple myoelectric control of a prosthesis^54^. Though our TLX scores decrease only slightly when the wrist is enabled, Hansen et al. showed that more intelligent and autonomous control strategies can reduce TLX scores to as low as 20–30^54^. Thus, there is still considerable room to improve the cognitive demands of functional wrists.

Amputees have expressed a strong desire for prostheses with a functional wrist^18^. The Utah wrist introduced here constitutes an important step towards meeting the needs of amputees as it demonstrates that low-cost systems can provide comparable benefits to high-end wrists. As a research tool, it will provide insight to the development of future prosthetic devices. The Utah wrist is inexpensive, adaptable to a variety of different terminal devices and prosthetic sockets, and, most importantly, was capable of significantly improving task performance when combined with a commercial prosthesis. Furthermore, the functional gain associated with adding the Utah wrist to the commercial prosthetic hand was similar to the functional gain seen enabling the commercial prosthetic arm’s embedded wrist.

Interestingly, not all participants preferred the research prosthesis configuration with the Utah wrist enabled over the research prosthesis configuration without the Utah wrist. This is despite the fact that they had a lower task failure rate and less compensation when using the Utah wrist. We speculate that one contributing factor to this discrepancy may be due to the fact that participants were blinded to the measurements of compensatory movements and were unaware that they produced substantially fewer compensatory movements with the research configuration with the Utah wrist enabled relative to the research configuration with the Utah wrist disabled. If participants were told to report preference outside the framework of an experimental test, preference may have favored the wrist conditions as in^1^. For example, the individual who rated R+W worse than R-W showed similar failure rates (37.5% and 40% respectively), but significantly less compensatory leftward bend (R-W: 19.0° vs. R+W: 10.3°, *p* < 0.05, unpaired *t*-test) and significantly less forward lean (R-W: 20.0° vs. R+W: 11.1°, *p* < 0.05, unpaired *t*-test). Because the participants had no feedback related to their compensatory movements, we speculate they simply selected the configuration with which they had the most attempted movements. Future work should consider providing amputees with real-time monitoring of compensatory movements, as the results presented here suggest prosthesis users are unlikely to be aware of their own compensatory movements during tasks. Within-task feedback may influence other subjective metrics as well, such as the TLX score.

The long-term user perception of an added wrist remains unknown. One confound of the present study is that simply disabling the wrist is not equivalent to removing the wrist, and weight is a critical priority for long-term use of a prosthesis^1^. Prior surveys of upper-limb amputees suggest they prioritize wrist function and hand function over weight^1^. However, the relative standard for weight was likely lower to begin with, as the survey respondents actively prioritizing wrist function are likely using lighter weight prostheses without a functional wrist. The added battery weight and/or reduction in battery life associated with a powered wrist may also limit long-term adoption of powered wrist prostheses. Similarly, the added length associated with a wrist may be prohibitive for individuals with long residual limbs; a lengthy prosthesis can make a prosthesis feel heavier and have a poorer socket fit.

An appropriate perceived length of the residual limb is also a critical aspect of embodiment^55^. Though embodiment was not specifically evaluated in this paper, future work could benefit from including a questionnaire to assess how well the inclusion of the wrist contributes to the embodiment of the device, or if embodiment played a role in the differences among the prostheses. The relationship between compensatory movements and embodiment is also unclear and should be investigated in the future.

Although use of a wrist here showed functional benefits, we note that addition of a wrist adds length and weight as well, which may impact overall performance and acceptance in real-world scenarios. Indeed, prior work has shown that weight and length are among top priorities among amputees^1^, and appropriate length has also been attributed to embodiment^56^. This study only explored use of a wrist in males with a left transradial amputation; future work should explore the impact of a wrist on females, who have shorter forearms than males on average, and the impact of hand dominance.

The results presented here highlight that the addition of a 2-DOF wrist does not significantly increase cognitive demand – and often trended towards reducing cognitive demand. This finding is substantial because it suggests that current myoelectric control strategies, such as the Modified Kalman Filter^25^, can readily accommodate additional controllable degrees of freedom. This in turn implies that the control strategies are not necessarily the primary bottleneck towards more widespread use of a functional prosthetic wrist. Instead, the bottleneck is more likely attributed to the slow translation of these new control strategies into the commercial space and mechanical and financial challenges associated with lightweight, compact, and robust designs. As noted previously, the Utah wrist is intended strictly for a research setting and is not intended to be a commercially used device.

Lastly, it is important to recognize that the modified CRT is a relatively simple task that does not fully capture the complexity of movements performed during activities of daily living. Nevertheless, we suspect that the added complexity of activities of daily living only further supports the need for a functional prosthetic wrist. As the number of take-home trials for advanced prostheses increases^57–60^, future work should explore the impact of a wrist on activities of daily living and broader quality of life metrics. There are a number of daily tasks that require wrist functionality^61^, and loss of wrist function has a major impact on quality of life^62^. Restoring functional wrist motion, as presented here, could have a positive impact on activities of daily living and quality of life.

Although the total unique participants in this study was rather small (*N* = 3), the data were collected in a repeated-measures design, increasing the reliability of the data and statistical power^63^. Further, an appropriate, multivariate statistical model was employed to analyze each outcome variable, accounting for the distribution of the variable and residuals, as well as for the correlated observations of the data. As a small-sample study is an important part of research in medicine and health^64,65^, we believe that the current study has uncovered important findings, with carefully designed study and analysis.

## Electronic supplementary material

Below is the link to the electronic supplementary material.

Supplementary Material 1

## Data Availability

The datasets used and/or analyzed during the current study available from the corresponding author on reasonable request.

## Abbreviations

ADL: Activities of daily living
CRT: Clothespin relocation task
DOF: Degrees of freedom
DRT: Detection response task
IMU: Inertial measurement unit
MAV: Mean absolute value
mKF: Modified Kalman filter
PLA: Polylactic acid
sEMG: Surface electromyography
TLX: Task load index

## References

[1] Atkins, D. J., Heard, D. C. Y. & Donovan, W. H. Epidemiologic overview of individuals with upper-limb loss and their reported research priorities. *J. Prosthet. Orthot.*** 8** (winter) (1996).

[2] Engdahl, S. M. et al. Surveying the interest of individuals with upper limb loss in novel prosthetic control techniques. *J. Neuroeng. Rehabil*. **12**, 53. 10.1186/s12984-015-0044-2 (2015). 26071402 10.1186/s12984-015-0044-2 PMC4465617

[3] Carey, S. L., Jason Highsmith, M., Maitland, M. E. & Dubey, R. V. Compensatory movements of transradial prosthesis users during common tasks. *Clin. Biomech.*** 23**(9), 1128–1135 10.1016/j.clinbiomech.2008.05.008 (2008). 10.1016/j.clinbiomech.2008.05.008 18675497

[4] Bertels, T., Schmalz, T. & Ludwigs, E. Objectifying the functional advantages of prosthetic wrist flexion. *JPO J. Prosthet. Orthot.*** 21**(2), 74–78. 10.1097/JPO.0b013e3181a10f46 (2009).

[5] Mell, A. G., Childress, B. L. & Hughes, R. E. The effect of wearing a wrist splint on shoulder kinematics during object manipulation, *Arch. Phys. Med. Rehabil.*** 86**(8), 1661–1664 10.1016/j.apmr.2005.02.008 (2005). 10.1016/j.apmr.2005.02.008 16084823

[6] Østlie, K., Franklin, R. J., Skjeldal, O. H., Skrondal, A. & Magnus, P. Musculoskeletal pain and overuse syndromes in adult acquired major upper-limb amputees. *Arch. Phys. Med. Rehabil*. **92** (12), 1967–1973. 10.1016/j.apmr.2011.06.026 (2011). 10.1016/j.apmr.2011.06.026 22133243

[7] LUKE Arm Detail Page – Mobius Bionics. https://www.mobiusbionics.com/luke-arm/. Accessed 1 Mar 2022 (2022).

[8] Johannes, M. S. et al. An overview of the developmental process for the modular prosthetic limb. *Johns Hopkins APL Tech. Dig.*** 30** (3), 10 (2011).

[9] Kyberd, P. J. et al. Two-degree-of-freedom powered prosthetic wrist. *J. Rehabil. Res. Amp. Dev.*** 48**(6), 609–618 10.1682/JRRD.2010.07.0137 (2011). 10.1682/jrrd.2010.07.0137 21938649

[10] Product. Wrist Rotator, Fillauer Motion Control. https://www.utaharm.com/product-wrist-rotator/. Accessed 15 Jun 2023 (2023).

[11] Electric Wrist Rotator. | Myo Wrist Units and Rotation | Myo Hands and Components | Upper Limb Prosthetics | Prosthetics | Ottobock US Shop. https://shop.ottobock.us/Prosthetics/Upper-Limb-Prosthetics/Myo-Hands-and-Components/Myo-Wrist-Units-and-Rotation/Electric-Wrist-Rotator/p/10S17. Accessed 15 Jun 2023 (2023).

[12] Roose, C. Two-degree-of-freedom pneumatically powered wrist prosthesis. https://repository.tudelft.nl/islandora/object/uuid%3A60cc243c-20d0-4ead-80d3-0bc29878e679. Accessed 6 Jun 2022 (2014).

[13] Mahmoud, R., Ueno, A. & Tatsumi, S. Dexterous mechanism design for an anthropomorphic artificial hand: Osaka City University Hand I. In *10th IEEE-RAS International Conference on Humanoid Robots*. 180–185 10.1109/ICHR.2010.5686843 (2010).

[14] Dange, S. V. Design of a working model of an upper limb prosthesis: wrist mechanism. *Rutgers Univ. - Sch. Grad. Stud.*10.7282/T30G3P8X (2017).

[15] Varley, E. & Joint, A. A. https://patentscope.wipo.int/search/en/detail.jsf?docId=WO2012098347. Accessed 6 June 2022 (2012).

[16] Razak, N. A. A., Osman, N. A. A., Gholizadeh, H. & Ali, S. Development and performance of a new prosthesis system using ultrasonic sensor for wrist movements: A preliminary study. *Biomed. Eng. OnLine*** 13** (1). 10.1186/1475-925X-13-49 (2014). 10.1186/1475-925X-13-49 PMC4014438 24755242

[17] Sex determination and estimation of stature from the long bones of the arm. *Forensic Sci. Int. Online*. **117**, 1–2. 10.1016/S0379-0738(00)00445-X (2001). 10.1016/s0379-0738(00)00445-x 11230943

[18] Safaee-Rad, R., Shwedyk, E., Quanbury, A. O. & Cooper, J. E. Normal functional range of motion of upper limb joints during performance of three feeding activities. *Arch. Phys. Med. Rehabil.*** 71**(7), 505–509 (1990). 2350221

[19] Kyberd, P., Hussaini, A. & Maillet, G. Characterisation of the clothespin relocation test as a functional assessment tool. *J. Rehabil. Assist. Technol. Eng.*** 5**, 2055668317750810. 10.1177/2055668317750810 (2018). 10.1177/2055668317750810 PMC6453097 31191921

[20] Hansen, T. C. et al. A multi-user transradial functional-test socket for validation of new myoelectric prosthetic control strategies. *Front. Neurorobot.* (online). https://www.frontiersin.org/articles/. 10.3389/fnbot.2022.872791/abstract (2022). 10.3389/fnbot.2022.872791 PMC9247306 35783364

[21] Williams, T., Meier, R. & Atkins, D. Control of powered upper extremity prostheses. *Funct. Restor. Adults Child. Up. Extrem Amput.*. **207**, 224 (2004).

[22] Pattern Recognition – Coapt, LLC, Coapt Myo Pattern Recognition. https://coaptengineering.com/pattern-recognition. Accessed 20 Aug 2024 (2024).

[23] Smith, L. H., Kuiken, T. A. & Hargrove, L. J. Real-time simultaneous and proportional myoelectric control using intramuscular EMG. *J. Neural Eng.*** 11** (6), 066013. 10.1088/1741-2560/11/6/066013 (2014). 10.1088/1741-2560/11/6/066013 PMC4268782 25394366

[24] George, J. A., Neibling, A., Paskett, M. D. & Clark, G. A. Inexpensive surface electromyography sleeve with consistent electrode placement enables dexterous and stable prosthetic control through deep learning. In *MEC20 Symposium*. https://conferences.lib.unb.ca/index.php/mec/article/view/36. Accessed 24 Sep 2020 (2020).

[25] George, J. A., Davis, T. S., Brinton, M. R. & Clark, G. A. Intuitive neuromyoelectric control of a dexterous bionic arm using a modified Kalman filter. *J. Neurosci. Methods*. **330**, 108462. 10.1016/j.jneumeth.2019.108462 (2020). 10.1016/j.jneumeth.2019.108462 31711883

[26] George, J. A., Radhakrishnan, S., Brinton, M. & Clark, G. A. Inexpensive and portable system for dexterous high-density myoelectric control of multiarticulate prostheses. In *IEEE International Conference on Systems, Man, and Cybernetics**(SMC)*. 3441–3446. 10.1109/SMC42975.2020.9283086 (2020).

[27] Paskett, M. D. et al. Activities of daily living with bionic arm improved by combination training and latching filter in prosthesis control comparison. *J. NeuroEng. Rehabil.*** 18**(1) 10.1186/s12984-021-00839-x (2021). 10.1186/s12984-021-00839-x PMC7908731 33632237

[28] George, J. A. et al. Robust torque predictions from electromyography across multiple levels of active exoskeleton assistance despite non-linear reorganization of locomotor output. *Front. Neurorobot.*. **15**, 700823. 10.3389/fnbot.2021.700823 (2021). 10.3389/fnbot.2021.700823 PMC8595105 34803646

[29] Wu, W., Gao, Y., Bienenstock, E., Donoghue, J. P. & Black, M. J. Bayesian population decoding of motor cortical activity using a Kalman filter. *Neural Comput.*** 18**(1), 80–118 10.1162/089976606774841585 (2006). 10.1162/089976606774841585 16354382

[30] Stojmenova, K. & Sodnik, J. Detection-response task-uses and limitations. *Sensors*. **18** (2). 10.3390/s18020594 (2018). 10.3390/s18020594 PMC5855461 29443949

[31] Guo, H., Boyle, L. N., Jenness, J. W. & Lee, J. D. Tactile detection response task: Metrics for assessing drivers’ cognitive workload. *Transp. Res. Part F Traffic Psychol. Behav.*** 70**, 98–108 10.1016/j.trf.2019.12.003 (2020).

[32] Accessed 14:00–17:00, ISO 17488:2016, ISO. https://www.iso.org/cms/render/live/en/sites/isoorg/contents/data/standard/05/98/59887.html. Accessed 15 June 2022 (2022).

[33] Čegovnik, T., Stojmenova, K., Jakus, G. & Sodnik, J. An analysis of the suitability of a low-cost eye tracker for assessing the cognitive load of drivers. *Appl. Ergon.*** 68**, 1–11. 10.1016/j.apergo.2017.10.011 (2018). 10.1016/j.apergo.2017.10.011 29409621

[34] Paskett, M. D. et al. Improving upper-limb prosthesis usability: Cognitive workload measures quantify task difficulty. *medRxiv*. 10.1101/2022.08.02.22278038 (2022).

[35] Trout, M. A. et al. Shared control decreases the physical and cognitive demands of maintaining a secure grip. In *Proceedings Myoelectric Controls Up. Limb Prosthetics Symposium* https://par.nsf.gov/biblio/10351635-shared-control-decreases-physical-cognitive-demands-maintaining-secure-grip. Accessed 5 June 2023 (2022). 10.57922/mec.1970 PMC13061088 41958759

[36] Buczak, M. K., Rosenbluth, J. & George, J. A. *Intuitive, Myoelectric Control of Adaptive Sports Equipment for Individuals with Tetraplegia*. 10.1109/ICORR58425.2023.10304759 PMC12795645 37941260

[37] Devos, H. et al. Psychometric properties of NASA-TLX and index of cognitive activity as measures of cognitive workload in older adults. *Brain Sci.*** 10**(12), 994 10.3390/brainsci10120994 (2020). 10.3390/brainsci10120994 PMC7766152 33339224

[38] McCullagh, P. *Generalized Linear Models*. 2nd edn 10.1201/9780203753736 (Routledge, 2019).

[39] Hardin, J. W. & Hilbe, J. M. *Generalized Linear Models and Extensions*. 2nd edn. (Stata Press, 2007).

[40] Huber, P. J. The behavior of maximum likelihood estimates under nonstandard conditions. In *Proceedings of the Fifth Berkeley Symposium on Mathematical Statistics and Probability*. , Vol. 1. Statistics. Vol. 5.1. 221–234. (University of California Press, 2024). https://projecteuclid.org/ebooks/berkeley-symposium-on-mathematical-statistics-and-probability/Proceedings-of-the-Fifth-Berkeley-Symposium-on-Mathematical-Statistics-and/chapter/The-behavior-of-maximum-likelihood-estimates-under-nonstandard-conditions/bsmsp/1200512988. Accessed 1 Apr 2024 (1967).

[41] White, H. Maximum likelihood estimation of misspecified models. *Econometrica*. **50** (1), 1–25. 10.2307/1912526 (1982).

[42] Minnotte, M. & James, J. Higgins introduction to modern nonparametric statistics. *Am. Stat.*, **61**, 2, 184–184. 10.1198/tas.2007.s81 (2007).

[43] Deijs, M., Bongers, R. M., Leusen, N. D. M. R. & van der Sluis, C. K. Flexible and static wrist units in upper limb prosthesis users: functionality scores, user satisfaction and compensatory movements. *J. Neuroeng. Rehabil*. **13** (27). 10.1186/s12984-016-0130-0 (2016). 10.1186/s12984-016-0130-0 PMC4791860 26979272

[44] van Kordelaar, J., van Wegen, E. E. H. & Kwakkel, G. Unraveling the interaction between pathological upper limb synergies and compensatory trunk movements during reach-to-grasp after stroke: A cross-sectional study. *Exp. Brain Res.*** 221**(3), 251–262 10.1007/s00221-012-3169-6 (2012). 10.1007/s00221-012-3169-6 PMC3412086 22791198

[45] Bloomer, C., Wang, S. & Kontson, K. Kinematic analysis of motor learning in upper limb body-powered bypass prosthesis training. *PLOS ONE*** 15**(1), e0226563 10.1371/journal.pone.0226563 (2020). 10.1371/journal.pone.0226563 PMC6980621 31978051

[46] Nguyen, G., Maclean, J. & Stirling, L. Quantification of Compensatory Torso Motion in Post-Stroke Patients Using Wearable Inertial Measurement Units, *IEEE Sens. J.*** 21**(13), 15349–15360. 10.1109/JSEN.2021.3072010 (2021).

[47] Major, M. J., Stine, R. L., Heckathorne, C. W., Fatone, S. & Gard, S. A. Comparison of range-of-motion and variability in upper body movements between transradial prosthesis users and able-bodied controls when executing goal-oriented tasks. *J. Neuroeng. Rehabil.*. **11** (1), 132. 10.1186/1743-0003-11-132 (2014). 25192744 10.1186/1743-0003-11-132 PMC4164738

[48] Kristoffersen, M. B. et al. User training for machine learning controlled upper limb prostheses: a serious game approach, *J. NeuroEng. Rehabil.*** 18**(1), 32 10.1186/s12984-021-00831-5 (2021). 10.1186/s12984-021-00831-5 PMC7881655 33579326

[49] Hussaini, A. & Kyberd, P. Refined clothespin relocation test and assessment of motion. *Prosthet. Orthot. Int.*** 41**(3), 294–302 10.1177/0309364616660250 (2017). 10.1177/0309364616660250 27473641

[50] Piazza, C., Rossi, M., Catalano, M. G., Bicchi, A. & Hargrove, L. J. Evaluation of a simultaneous myoelectric control strategy for a multi-DoF transradial prosthesis. *IEEE Trans. Neural Syst. Rehabil. Eng.*** 28**(10), 2286–2295 10.1109/TNSRE.2020.3016909 (2020). 10.1109/TNSRE.2020.3016909 PMC9028175 32804650

[51] Crea, S., Edin, B. B., Knaepen, K., Meeusen, R. & Vitiello, N. Time-discrete vibrotactile feedback contributes to improved gait symmetry in patients with lower limb amputations: Case series. *Phys. Ther.*** 97** (2), 198–207. 10.2522/ptj.20150441 (2017). 10.2522/ptj.20150441 28204796

[52] Gonzalez, J., Soma, H., Sekine, M. & Yu, W. Psycho-physiological assessment of a prosthetic hand sensory feedback system based on an auditory display: A preliminary study. *J. Neuroeng. Rehabil*. **9** (1), 33. 10.1186/1743-0003-9-33 (2012). 10.1186/1743-0003-9-33 PMC3481360 22682425

[53] O’Meara, S. M., Shyr, M. C., Lyons, K. R. & Joshi, S. S. Comparing two different cursor control methods which use single-site surface electromyography. In *9th International IEEE/EMBS Conference on Neural Engineering (NER)*. 1163–1166. 10.1109/NER.2019.8716903 (2019).

[54] Hansen, T. C., Trout, M. A., Segil, J. L., Warren, D. J. & George, J. A. A bionic hand for semi-autonomous fragile object manipulation via proximity and pressure sensors. *Annu. Int. Conf. IEEE Eng. Med. Biol. Soc. IEEE Eng. Med. Biol. Soc. Annu. Int. Conf.*. 6465–6469 10.1109/EMBC46164.2021.9629622 (2021). 10.1109/EMBC46164.2021.9629622 PMC12742981 34892591

[55] Longo, M. R., Long, C. & Haggard, P. Mapping the invisible hand: A body model of a phantom limb, *Psychol. Sci.*** 23**(7), 740–742 10.1177/0956797612441219 (2012). 10.1177/0956797612441219 PMC3494453 22653797

[56] Engdahl, S. M., Meehan, S. K. & Gates, D. H. Differential experiences of embodiment between body-powered and myoelectric prosthesis users. *Sci. Rep.*** 10**(1), Art. no. 1 10.1038/s41598-020-72470-0 (2020). 10.1038/s41598-020-72470-0 PMC7508812 32963290

[57] An osseointegrated human-machine gateway for long-term sensory feedback and motor control of artificial limbs. https://www.science.org/doi/epdf/. 10.1126/scitranslmed.3008933. Accessed 7 Jun 2022 (2022). 10.1126/scitranslmed.3008933 25298322

[58] Earley, E. J. et al. Competitive motivation increased home use and improved prosthesis self-perception after Cybathlon 2020 for neuromusculoskeletal prosthesis user. *J. NeuroEng. Rehabil.*** 19**(1) 10.1186/s12984-022-01024-4 (2022). 10.1186/s12984-022-01024-4 PMC9112467 35578249

[59] Osborn, L. E. et al. Extended home use of an advanced osseointegrated prosthetic arm improves function, performance, and control efficiency. *J. Neural Eng.*** 18**(2), 026020 10.1088/1741-2552/abe20d (2021). 10.1088/1741-2552/abe20d 33524965

[60] Graczyk, E. L., Resnik, L., Schiefer, M. A., Schmitt, M. S. & Tyler, D. J. Home use of a neural-connected sensory prosthesis provides the functional and psychosocial experience of having a hand again. *Sci. Rep. Nat. Publ. Group*** 8**, 1–17. 10.1038/s41598-018-26952-x (2018). 10.1038/s41598-018-26952-x PMC6026118 29959334

[61] Moser, N., O’Malley, M. K. & Erwin, A. Importance of wrist movement direction in performing activities of daily living efficiently. *Annu. Int. Conf. IEEE Eng. Med. Biol. Soc. IEEE Eng. Med. Biol. Soc. Annu. Int. Conf.*. 3174–3177 10.1109/EMBC44109.2020.9175381 (2020). 10.1109/EMBC44109.2020.9175381 33018679

[62] Puranik, A. K. et al. Quality of life of patients with major amputations in the Tertiary Care Center of Western Rajasthan: A prospective observational study in 2019–2020. *Cureus*** 13**(12), e20419. 10.7759/cureus.20419 10.7759/cureus.20419 PMC8671130 34926097

[63] The Power of Replicated Measures to Increase Statistical Power - Marc-André & Goulet Denis Cousineau, 2019. https://journals.sagepub.com/doi/full/10.1177/2515245919849434. Accessed 1 Apr 2024 (2024).

[64] Lillie, E. O. et al. The n-of-1 clinical trial: The ultimate strategy for individualizing medicine? *Pers. Med.*** 8**(2), 161–173. 10.2217/pme.11.7 (2011). 10.2217/pme.11.7 PMC3118090 21695041

[65] *The Essential Guide to N-of-1 Trials in Health*. https://link.springer.com/book/10.1007/978-94-017-7200-6. Accessed 1 Apr 2024 (2024).

