# Supplementary material for: Electromyographically controlled prosthetic wrist improves dexterity and reduces compensatory movements without added cognitive load
Source: Sci Rep. 2024 Oct 6;14:23248. doi: 10.1038/s41598-024-73855-1 (PMC11456584; doi:10.1038/s41598-024-73855-1)
Supplement: Supplementary file 1 — Supplementary Material 1 [file 41598_2024_73855_MOESM1_ESM.docx]

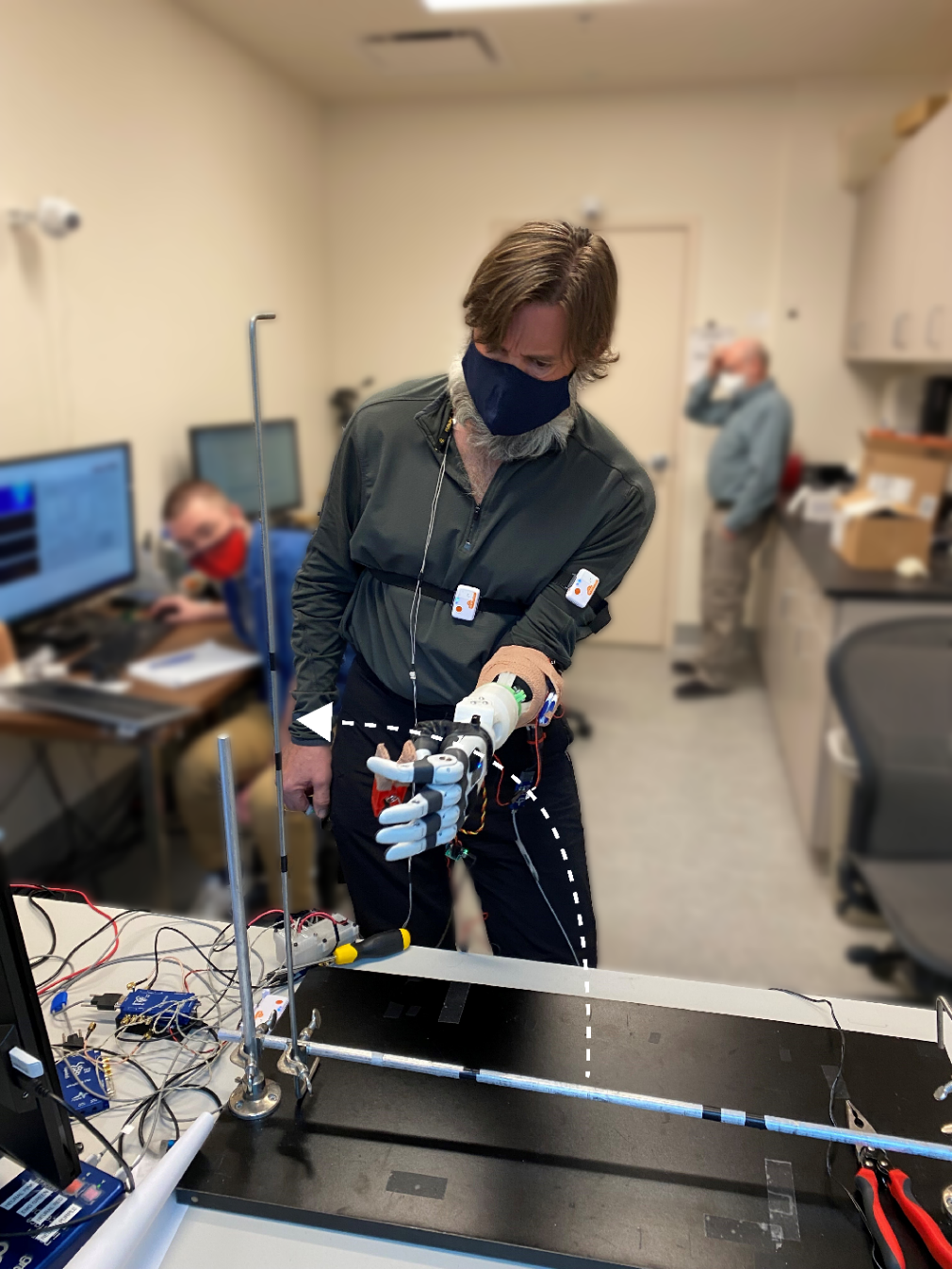


**Supplementary Figure S1** Photograph depicting the modified clothespin relocation task, moving the clothespin from the horizontal bar to the vertical bar. Note the white IMUs placed on the chest and arm.


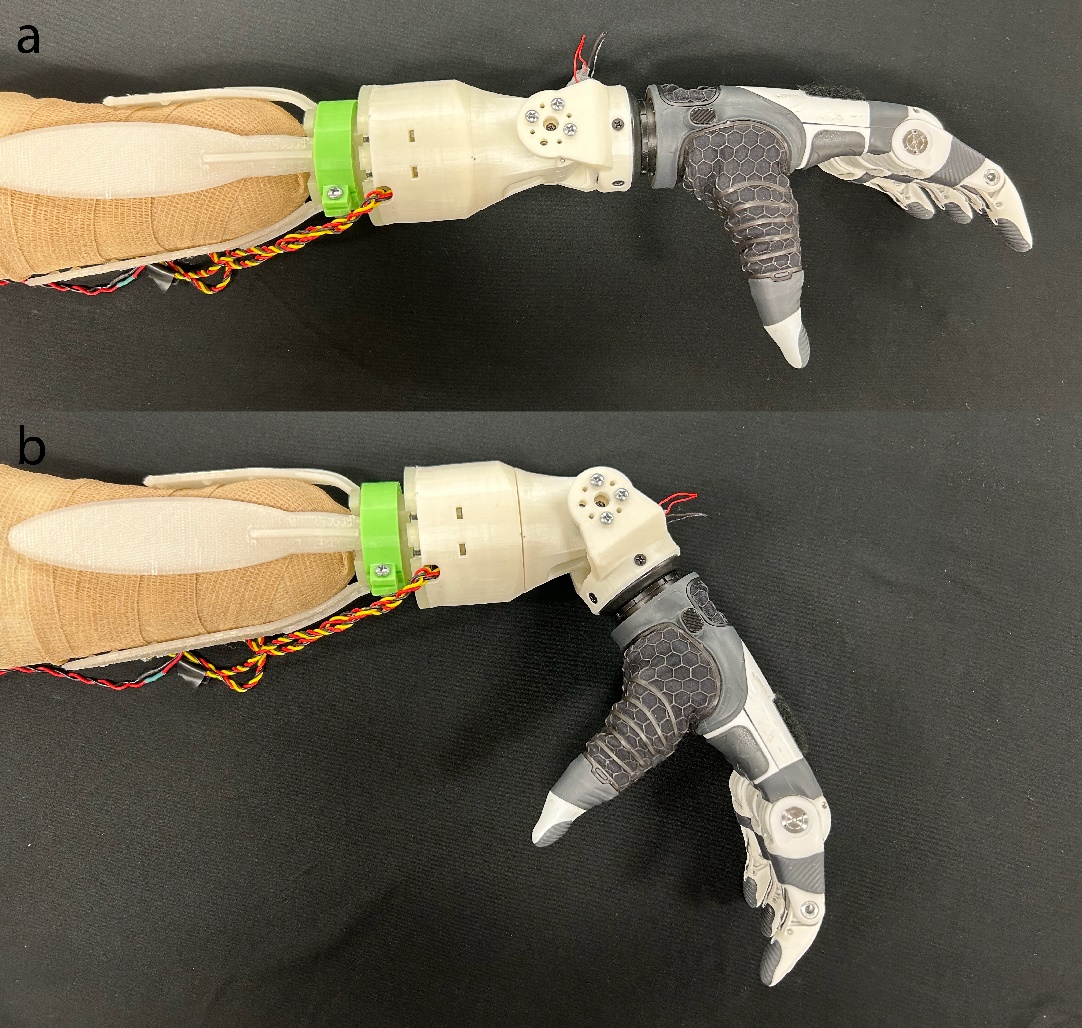


**Supplementary Figure S2** Research configuration consisting of the TASKA Hand connected to the Utah Wrist. **a** The research configuration attached to the multi-user functional check socket. **b** The research configuration with the wrist in a flexed position.


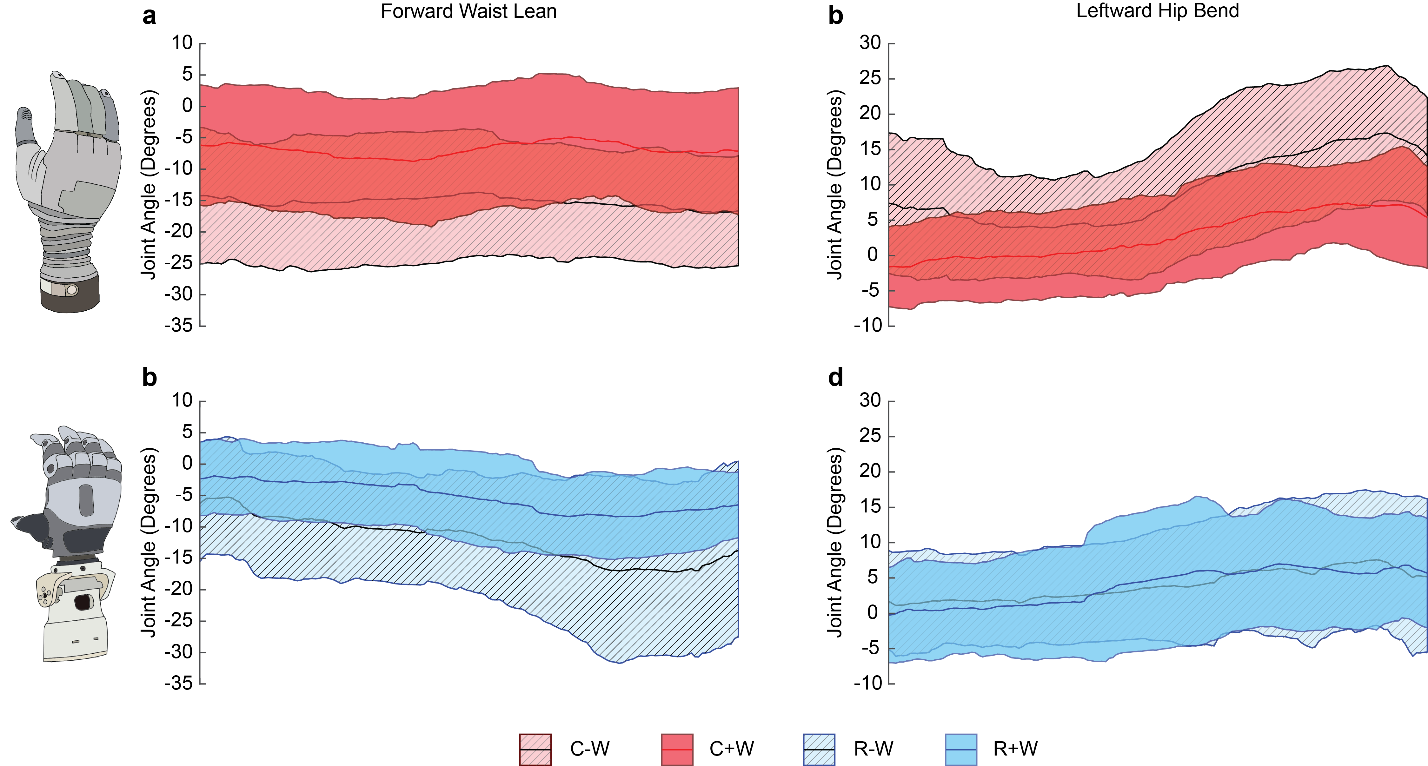


**Supplementary Figure S3** Average joint angles over time. **a** Forward waist lean compensatory movement for the commercial configuration. **b** Forward waist lean compensatory movement for the research configuration. **c** Leftward hip bend compensatory movement for the commercial configuration. **d** Leftward hip bend compensatory movement for the research configuration.

**
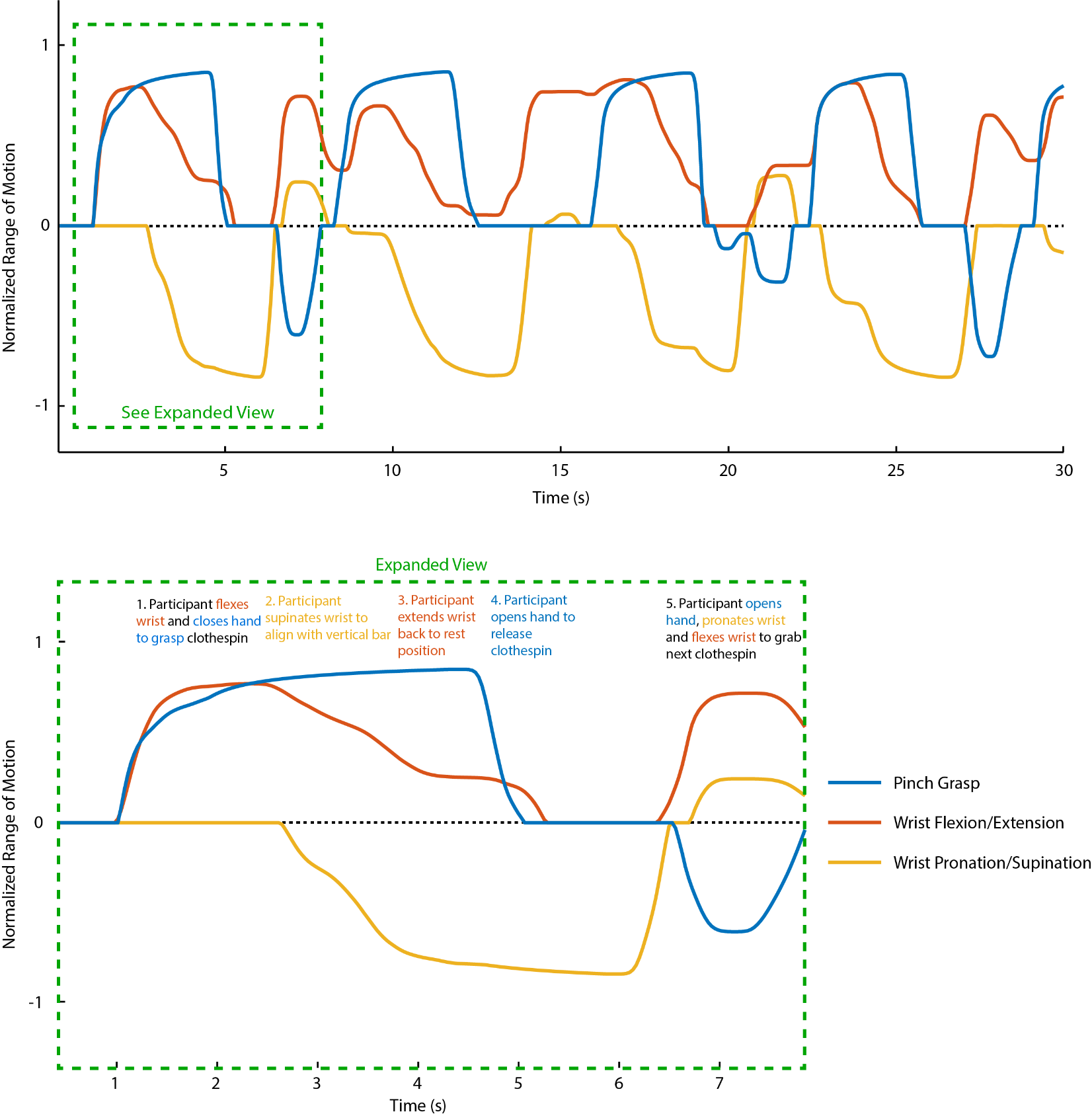
****Supplementary Figure S4** Example traces of the different degrees of freedom while a participant completed one trial of the CRT in the C+W configuration. The expanded view shows one attempt in which the participant first flexes their wrist and grabs the clothespin, then supinates to align with the vertical bar, and finally opens the hand and returns to the neutral position to grab the next clothespin. Traces show normalized range of motion, such that +1 equates to maximum hand grasping, maximum wrist flexion, or maximum wrist pronation, 0 equates to a neutral resting position, and -1 equates to maximum hand opening, maximum wrist extension, or maximum wrist supination.


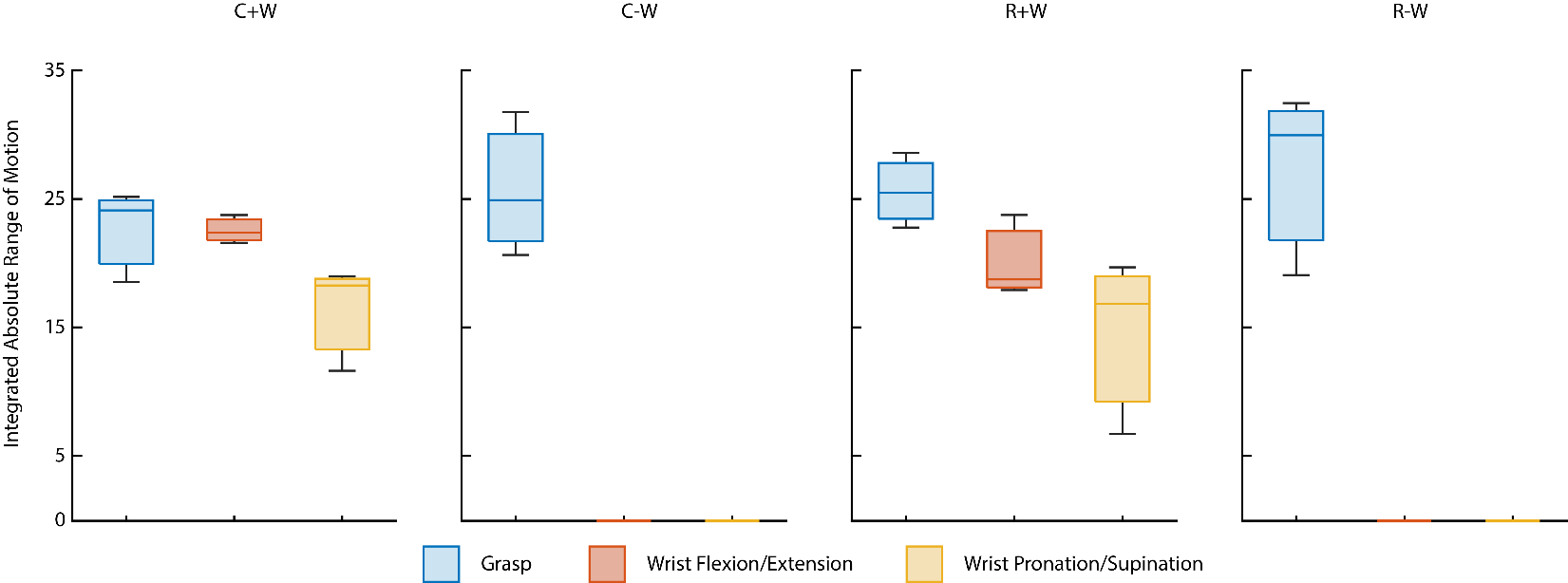
**Supplementary Figure S5** Relative use of each controllable DOF. Data show the integrated absolute range of motion across all attempts. Boxplots show the median, interquartile range, and most extreme non-outlier values. N = 3 participants.

**Supplementary Table S1** Summary of analysis on outcome variables by C+W vs. C-W

**Supplementary Table S2** Summary of analysis on outcome variables by R+W vs. R-W
